# Fanaticism

**Published:** 1849-12

**Authors:** 


*Fanaticism.*—Dr. Martin, of Hinsdale, N. H., has been fined $75 for violation of the license law of the state. He sold the spirits as a medicine, being himself a *temperance man!*— *Western Lancet.*

